# p53 aberration and *TFE3* gene amplification may be predictors of adverse prognosis in epithelioid angiomyolipoma of the kidney

**DOI:** 10.1186/s13000-023-01298-9

**Published:** 2023-02-06

**Authors:** Yanning Zhang, Xuejing Wei, Xiaojing Teng, Guangyong Chen

**Affiliations:** Department of Pathology, Beijing Friendship Hospital, Capital Medical University, No. 95 Yong-an Road, Xicheng District, Beijing, 100050 China

**Keywords:** Epithelioid angiomyolipoma, Kidney, p53, PEComa, *TFE3* gene

## Abstract

**Background:**

Although epithelioid angiomyolipoma of the kidney has been studied by several groups, the reported prevalence of malignant behavior remains uncertain and there are not yet definitive predictive biomarkers. We evaluated the behavior of renal epithelioid angiomyolipoma in a consecutive series in a single institution and investigated the prognostic value of aberrant p53 expression and *TFE3* gene abnormality.

**Methods:**

We retrospectively reviewed 14 epithelioid angiomyolipomas, most with pure or close to pure epithelioid components, comprising 12 consecutive cases who had attended our institution and two consultation cases. Fluorescence in situ hybridization with *TFE3* break-apart probe was performed on 14 cases. The 14 cases were also labeled for p53 and TFE3 by immunohistochemistry. All cases were followed up.

**Results:**

Three of the epithelioid angiomyolipomas were strongly positive for TFE3 and two had a mutant expression of p53. Although no *TFE3* gene rearrangement was found, the two tumors with strong TFE3 expression showed *TFE3* gene amplification. Follow-up details were available for seven of the 12 consecutive cases: two of them had developed metastases and died (29%), their mean overall survival was 41 months, and both had mutant p53 expression. The two consultation cases with *TFE3* gene amplification developed recurrence/metastasis within 1 year after surgery.

**Conclusions:**

Our series study from a single institution presented the prevalence of malignant behavior in pure epithelioid angiomyolipomas, although the small number of cases with follow-up data greatly reduced the accuracy. p53 may be a prognostic marker for epithelioid angiomyolipoma. Cases with *TFE3* gene amplification had poor prognoses.

## Introduction

Epithelioid angiomyolipoma (epithelioid-AML) of the kidney is a member of the perivascular epithelioid cell tumor (PEComa) family [1]. In the 2016 World Health Organization Classification of Renal Neoplasms, epithelioid-AML of the kidney is defined as a rare variant of AML that consists of at least 80% epithelioid cells with borderline biological behavior [2]; it is also called PEComa of the kidney. Because of differences in the percentage of epithelioid cells and types of cases included (consecutive or consultation) in different studies, the reported prevalence of malignant biological behavior is extremely variable and inconsistent, ranging from 5 to 66% [3–8]. Hence, it is necessary to reevaluate the biological behavior of renal epithelioid-AML in a way that avoids these differences which may introduce bias in prevalence. p53 is the protein encoded by the tumor suppressor gene, *TP53*. Mutant p53 expression is seen in many malignant tumors. To date, the prognostic value of mutant p53 expression in epithelioid-AML of the kidney has not been investigated. Some renal epithelioid-AMLs with *TFE3* gene abnormalities reportedly exhibit aggressive biological behavior; thus, *TFE3* gene abnormalities seem to be correlated with poor prognoses. To investigate the prognostic significance of mutant p53 expression and *TFE3* gene abnormality, we studied 14 cases of renal epithelioid-AML, 12 of which were consecutive cases from a single institution, they were analyzed with emphasis on biological behavior.

## Materials and methods

### Case selection

After approval by the Institutional Ethics Committee (No: 2022-P2-004-01), the surgical pathology files of the Department of Pathology, Beijing Friendship Hospital affiliated with Capital Medical University were searched electronically for patients with renal epithelioid-AML. Three hundred and thirty-six consecutive cases of AML of the kidney resected and diagnosed at Beijing Friendship Hospital from January 2011 to October 2021 were retrieved. Twelve of them were diagnosed as epithelioid-AMLs on the basis of the following criteria: (i) the neoplasm was primarily located in the kidney; (ii) the histomorphology was of AML comprising at least 80% epithelioid neoplastic cells; (iii) the neoplastic cells did not express the epithelial markers cytokeratin (AE1/AE3) or epithelial membrane antigen (EMA) and did not express PAX8; (iv) the neoplastic cells expressed actins (smooth muscle actin or calponin); if they did not express actin, the renal epithelial-related marker PAX8 had to be negative; and (v) the neoplastic cells expressed at least one melanocytic marker, such as HMB-45 or Melan-A. Two patients with renal epithelioid-AML were referred to our institution for a second opinion (consultation cases) during the study period, hence they were included in this study. All 14 cases were reviewed and confirmed by two pathologists.

### Immunohistochemistry

Immunohistochemistry was performed on sections from the 14 cases of epithelioid-AML with the following antibodies: p53 (ready-to-use, Roche; high-grade serous carcinoma and tonsil tissue was used as positive control), TFE-3 (ready-to-use, Maxim), actin (ready-to-use, Maxim), HMB-45 (1:1000, Maxim), Melan-A (1:100, Maxim), S-100 (1:200, Maxim), SOX-10 (ready-to-use, ZSGB Biotechnology), AE1/AE3 (1:200, Maxim), epithelial membrane antigen (1:100, Maxim), vimentin (1:200, Maxim), carbonic anhydrase IX (1:100, Maxim), PAX8 (1:80, Maxim), Ki-67 (1:200, Maxim), desmin (1:200, Maxim), and cathepsin K (1:400, Abcam).

### Fluorescence in situ hybridization

Using dual color *TFE3* break-apart probe (Guang Zhou LBP Medicine Science and Technology), fluorescence in situ hybridization (FISH) was carried out on archival material from the 14 studied cases of renal epithelioid-AML. This probe set includes a 5′*TFE3* centromeric-direction probe, labeled in spectrum green, and a 3′*TFE3* telomeric-direction probe, labeled in spectrum orange, which gives a red signal. The proximity of red and green signals in non-rearranged *TFE3* results in a normal yellow or red–green combined signal. Cases 13 and 14 were also subjected to additional hybridization using an X centromere probe, which labels the chromosome X centromere. The protocols for pretreatment, hybridization, and post-hybridization washes were essentially performed as recommended by the manufacturer. *TFE3* rearrangement results in a split signal in which the distance between the red and green signals is greater than twice the signal diameter. One hundred, randomly selected, non-overlapping, neoplastic cell nuclei were counted, the positive cut-off for *TFE3* rearrangement being that at least 10% of neoplastic cells had split signals. The presence of more than two yellow/red–green combined signals in nearly every neoplastic cell that showed 1 (male) or 2 (female) centromeric signals, was considered to denote amplification. Scoring was performed by one specialized molecular pathologist and one urological pathologist.

### Follow up

All study cases were followed up. The follow-up data of the consultation cases and consecutive cases were analyzed separately.

Clinical information was collected from the hospital’s medical records and the patients were followed up through phone calls. The following clinical characteristics were recorded: age, symptoms, treatment, and current status (alive with tumor, alive with no evidence of tumor, recurrence, metastasis, death).

### Statistical analysis

Statistical analysis was carried out using the Statistical Package for the Social Sciences software version 22.0. The relationship between p53 aberration (mutant expression) and the prognosis of the 12 consecutive cases resected at our institution were analyzed using Fisher’s exact test. A value of p<0.05 was considered to denote statistical significance.

## Results

In this study, the incidence of renal epithelioid-AML among all consecutively resected AML samples (336 cases) was 3.6%. The mean age at presentation was 40 years (range 30–56 years) with a female: male ratio of 7:7. None of the patients had tuberous sclerosis complex. Seven of the 14 patients had been found to have a renal tumor during a physical examination; four had persistent discomfort or pain at the waist level; one anemia and intermittent fever; and two abdominal discomforts before the tumor was found and treated. Seven patients underwent partial nephrectomy, four total nephrectomy, two unilateral radical nephrectomy with adrenal gland, and one adrenalectomy 3 years after the nephrectomy. There were no lymph node dissections or other concurrent renal tumors in any of the 14 cases. The margins were free of tumors in all cases.

The tumor size ranged from 1.5–20 cm (mean 7.11 cm). Two tumors were completely solid whereas the other twelve had cysts of varying size that contained areas of hemorrhage. Macroscopically, the tumor sections were mainly gray and brown.

The main pathological features of the 14 cases are summarized in Table 1. Most of the cases (13/14) were pure (100%) or close to pure (98%) epithelioid-AMLs. Six of the tumors showed a completely carcinoma-like pattern, three a pure diffuse pattern with epithelioid and plump spindle cells, the remaining five having varying proportions of carcinoma-like and diffuse growth patterns. Eleven of the fourteen epithelioid-AMLs contained malformed blood vessels, twelve perivascular growth structures, four focal acinar structures, and one melanin pigment.

**Table 1:** Detailed clinicopathological features and clinical outcomes of 14 cases of renal epithelioid angiomyolipoma

Case	Age	Sex	Tumor size (cm)	Epithelioid cells (%)	Growth pattern	Significant atypia	MGN cells (%)	Perivascular growth	Surgical method
1	52	F	4.5	100	A	Diffuse	5	Present	total nephrectomy
2	43	M	4.6	100	A&B	Diffuse	20	Present	partial nephrectomy
3	43	M	5.5	100	A&B	Focal	5	Present	partial nephrectomy
4	43	F	9	98	A	Diffuse	30	Present	total nephrectomy with adrenalectomy
5	34	M	12	100	A	Focal	5	Present	total nephrectomy
6	43	F	1.5	100	B	Focal	0	absent	partial nephrectomy
7	35	F	20	100	A	Diffuse	40	Present	total nephrectomy, followed by adrenalectomy
8	31	M	2.4	98	B	Focal	10	Present	partial nephrectomy
9	56	M	3	100	A&B	Diffuse	5	Present	partial nephrectomy
10	30	M	9.5	100	A&B	Focal	10	Present	partial nephrectomy
11	36	F	8	98	A&B	Focal	10	Present	total nephrectomy
12	32	M	3.5	80	B	Focal	5	Present	partial nephrectomy
13 ^c^	36	F	4.5	100	A	Diffuse	40	absent	total nephrectomy with adrenalectomy
14 ^c^	43	F	11.5	100	A	Diffuse	40	Present	total nephrectomy
total	mean:40	F,7; M,7	mean:7.11	mean:98	B: 3; A: 6; A&B: 5	Present: 14 /14 Diffuse,7; focal,7	Range:0–40 Mean:17	Present: 12 /14 (86%)	total: 4 /14 partial: 7 /14 adrenal-nephrectomy:3/14
Case	Tumor necrosis	Malformed blood vessels	Mitosis/50HPF	p53 expression	TFE-3 staining	FISH (*TFE3*)	Local invasion	Follow up (month)	Outcome
1	No	absent	<1	Wild	(−)	Normal	No	66	Alive, NET
2	Yes	Present	<1	Wild	(−)	Normal	No	unavailable	NA
3	No	Present	<1	Wild	(−)	Normal	No	unavailable	NA
4	Yes	Present	14^a^	mutant	(+, strong)	Normal	adrenal	10	Metastasis and dead
5	Yes	Present	<1	Wild	(−)	Normal	No	84	Alive, NET
6	No	Present	<1	Wild	(−)	Normal	No	unavailable	NA
7	Yes	Present	4^a^	mutant	(−)	No signal	adrenal	72	Metastasis and dead
8	No	Present	<1	Wild	(−)	Normal	No	4	Alive, NET
9	Yes	Present	<1	Wild	(−)	Normal	perinephric fat tissue	10	Alive, NET
10	No	Present	<1	Wild	(−)	Normal	perinephric fat tissue	19	Alive, NET
11	No	Present	<1	Wild	(−)	Normal	No	unavailable	NA
12	No	Present	<1	Wild	(−)	Normal	No	unavailable	NA
13 ^c^	Yes	absent	9^a^	Wild	(+, strong)	Amplification	perinephric fat tissue	7	Recurrence
14 ^c^	Yes	absent	9^a^	Wild	(+, strong)	Amplification	Renal sinus	8	Metastasis
total	Present: 7 /14 (50%)	Present: 11 /14 (79%)	<1: 10; 4–14: 4		Positive-rate :3/14 (21%)	“Split” rate: 0% (0/13)	Local invasion: 6/14 (42.9%)	Range:4–84	

*Abbreviations: A* carcinoma-like pattern, *B* diffuse growth pattern, *NA* not available, *(−) *negative, *(+) *positive, *MGN* Multinucleated giant neoplastic cells, *NET* no evidence of tumor, ^a^ Atypical mitoses present, ^c^ consultation cases

In all cases, the neoplastic cells were large and polygonal with rich eosinophilic cytoplasm and large red nucleoli. Significant atypia was seen in all 14 cases; it was diffuse (≥ 50%) in seven and focal (< 30%) in the other seven. Geographic tumor necrosis was present in seven cases. Multinucleated giant tumor cells scattered singly or in small clusters were seen in most cases. There were areas of clear cells in nine cases; these closely resembled clear cell carcinoma. Ten cases had < one mitosis/50 high power fields (HPFs) and the remaining four cases > one mitosis/50 HPFs; pathological mitoses were present in the latter.

**Fig. 1:**
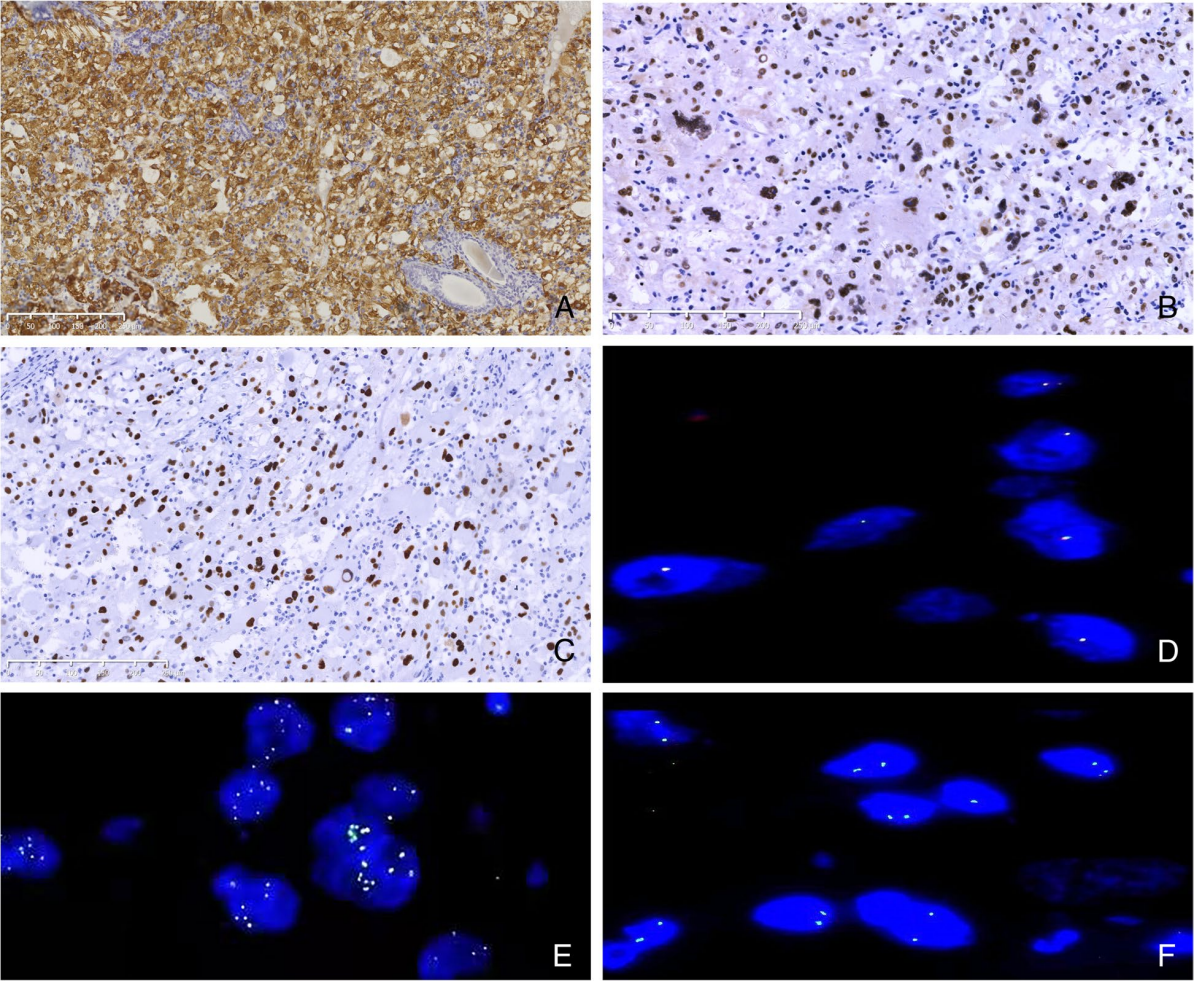
The main abnormality of immunohistochemistry (IHC) staining and Fluorescence in situ hybridization (FISH) results. **A** All tumor cells were positive for HMB45 with IHC staining; **B** TFE3 was strongly positive in tumor nuclei with IHC staining; **C** p53 was strongly positive in all tumor cell nuclei with mutant expression pattern; **D** FISH analysis of *TFE3* gene break, cases 5 (male) showed single yellow or red-green combined signal in neoplastic nuclei, indicating no evidence of *TFE3* gene rearrangement; **E** *TFE3* break-apart FISH image revealed that *TFE3* amplification exists in cases 13 in which there are 3 to 8 copies of combination signals found in each neoplastic nucleus; **F** FISH with X centromere probe labeled 2 X chromosome centromeric signals in each neoplastic nucleus in cases 13 (female) which showed *TFE3* gene amplification

All 14 renal epithelioid-AMLs strongly and diffusely expressed HMB-45 (Fig. 1A) and all were negative for AE1/AE3. Melan-A (Mart-1) was expressed in 11/11 cases, cathepsin K in 13/14, and actin in 12/14 cases. TFE3 was strongly expressed in three of 14 cases (Fig. 1B; Cases 4, 13, and 14). p53 showed a mutant immunostaining pattern (overexpression or complete loss) in 2/14 cases (Fig. 1C; Cases 4 and 7). Ki-67 proliferation index was ≤1% in eight cases, 5% in two, 10% in one, and 20% in three. Carbonic anhydrase IX was negative in 6/6 cases and PAX-8 was negative in 7/7 cases. Desmin was negative in 6/6 cases. S-100 was negative in 8/8 cases.

In the 12 consecutive cases, none of 11 cases showed split or amplified *TFE3* fluorescent signals (Fig. 1D); one case showed no signal even after repeating hybridization twice, possibly because the paraffin block was too old for the *TFE3* gene to be detectable. The two consultation cases (Cases 13 and 14, both female) showed *TFE3* gene amplification: three to eight *TFE3* probe signals (Fig. 1E) were present in each neoplastic nucleus in which normal 2 X chromosome centromeric signals were displayed (Fig. 1F) with a X centromere probe. No red–green split signals were found in the two consultation cases.

**Fig. 2:**
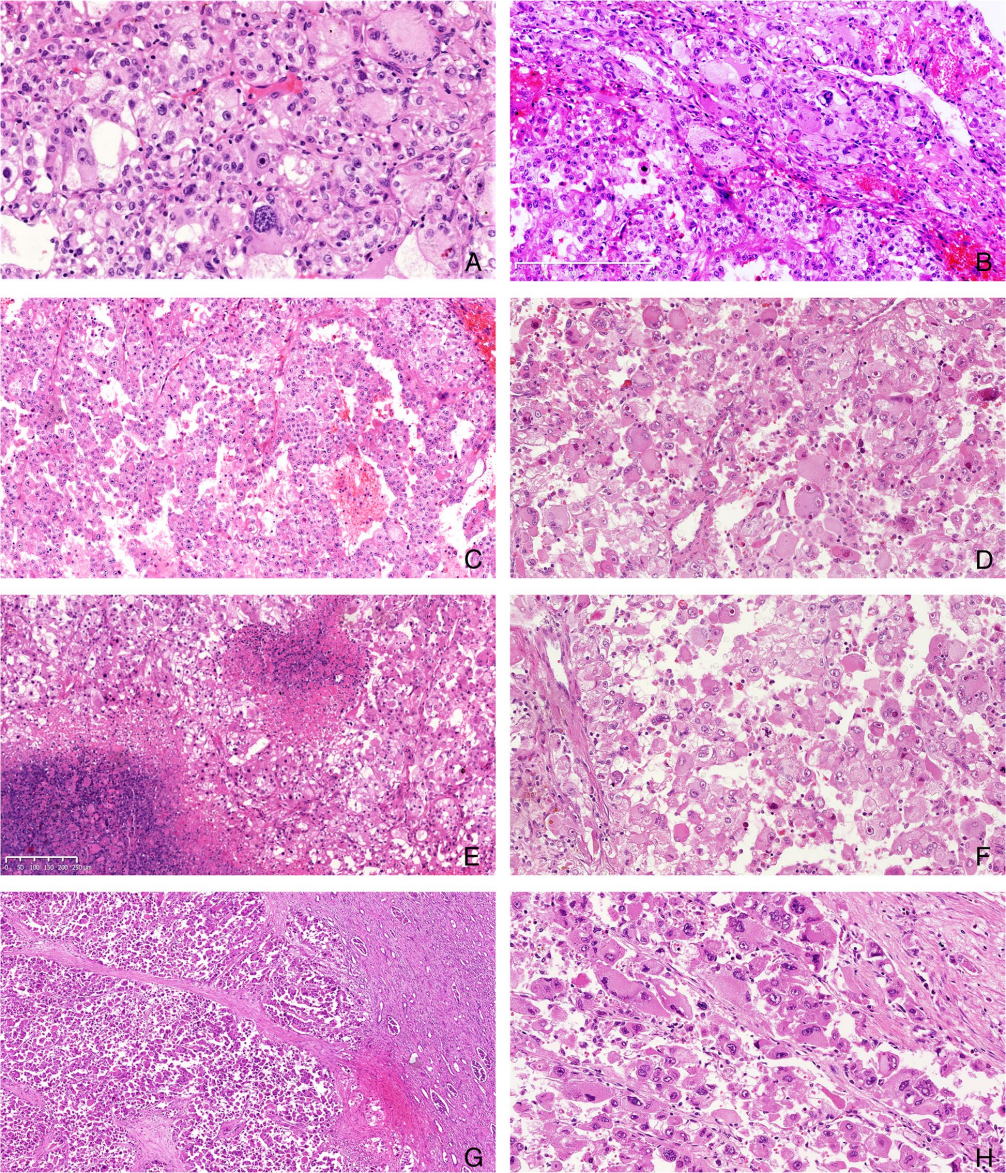
Morphological features of renal epithelioid angiomyolipoma with recurrence/metastasis in hematoxylin-eosin (H&E) staining. **A** and **B** Carcinoma-like pattern, the tumor cells were arranged as cohesive nests separated by thin vascular-rich or fibrous septa; **C** Pseudoglandular configurations were present focally due to discohesive cells in the center; **D** Multinucleate giant tumor cells with similar nuclear features to background neoplastic cells were present in clusters; **E** Geographic tumor necrosis; **F** Pathological mitosis (in the center of image); **G** The neoplastic cells were diffusely discohesive; **H** The neoplastic cells were large with diffusely significant atypia: cytoplasm was abundant, dense and deeply eosinophilic, the nuclei were hyperchromatic and pleomorphic, and nucleoli were highly prominent

To avoid selection bias of unusual cases with unusual clinical behavior, the follow-up data of the two consultation cases were not analyzed with the 12 consecutive cases. Follow-up data were available for seven of the 12 consecutive cases (Table 1), their follow-up time ranging from 4 to 84 months (mean 37.8 months). Five of these patients survived tumor-free and two (2/7, 29%) died of metastases, which were in the lungs in both cases. In addition to mutant p53 expression, the two cases with metastases (Cases 4 and 7) had the following features (Table 1): larger tumor size, local invasion, complete carcinoma-like pattern (Fig. 2A, B), focal pseudoglandular structures (Fig. 2C), diffuse significant atypia, a large percentage of multinucleated giant tumor cells (Fig. 2D), tumor necrosis (Fig. 2E), high mitotic count (> one mitosis/50 HPF), and pathological mitosis (Fig. 2F). These two patients survived for 10 and 72 months. The mean overall survival time was 41 months. The relationship between mutant p53 expression and prognosis of the consecutive cases was analyzed using Fisher’s exact test. Univariate analysis (Fisher’s exact two-sided test) showed that p53 aberration was correlated with a poor prognosis (*p* < 0.05).

The two consultation cases both had *TFE3* amplification and recurrence/metastasis. One of the tumors recurred locally 7 months postoperatively and the other one had recurrence and metastasis 8 months postoperatively, the site of metastasis being the greater omentum. The two consultation cases (Cases 13 and 14) were found to exhibit the following features: local invasion, complete carcinoma-like pattern, discohesive tumor cells (Fig. 2G), diffuse significant atypia (Fig. 2H), a large percentage of multinucleated giant tumor cells, tumor necrosis, a high mitotic count (> one mitosis/50 HPF), and pathological mitosis.

## Discussion

Since Martignoni et al. first reported renal epithelioid-AML [9], many of these tumors have been reported worldwide. Similar to previous studies [7], the mean age of our study patients was 40 years and the sex proportion was even. The incidence of renal epithelioid-AML among patients with AML reportedly varies from 3.9 to 7.7% [8, 10–12]. The 12 consecutive cases with predominant or pure epithelioid components from our institution that we reviewed accounted for 3.6% of all resected renal AMLs. Given that all of our patients were Chinese, this may be an epidemiological characteristic of epithelioid-AML in Chinese population.

Before 2016, the percentage of epithelioid cells required for the diagnosis of renal epithelioid-AML was not defined and varied from 5 to 100% [3–6], several reports not even specifying the percentage of epithelioid cells. This inconsistency in diagnostic criteria for renal epithelioid-AML led to discrepancies between the conclusions drawn in different studies. This probably accounts for the dramatic variation, from 5 to 66%, in the prevalence of malignant behavior of renal epithelioid-AML reported by several large studies [3, 4, 7, 8]. Considering these data, the behavior of renal epithelioid-AML is defined by the WHO Classification of Renal Neoplasms as borderline or uncertain behavior. Two relatively large series of renal epithelioid-AMLs with a pure or at least 80% epithelioid component (Nese et al. and He et al.) have reported conflicting results [7, 8]. Whereas metastases and death occurred in 49% and 33% of patients, respectively, in the study by Nese et al. [7], He et al. reported that only 5% of renal epithelioid-AMLs showed malignant behavior [8]. The 41 cases analyzed by Nese et al. were all pure epithelioid-AMLs and included consultation cases which may have caused a bias of selection of unusual cases. He et al.’s study did not include consultation cases; however, a considerable proportion of their cases in the study were not pure epithelioid-AMLs. To avoid differences related to the percentage of epithelioid cells and types of selected cases, such as consecutive versus consultation cases, that may cause bias, our study cohort comprised 12 consecutive cases, most of which had pure or close to pure epithelioid components, drawn from 336 consecutive cases of renal AML in one institution. Because of its rarity, most reports of pure epithelioid-AMLs of the kidney have been in the form of case reports. To the best of our knowledge, this is the second published series of pure renal epithelioid-AMLs, the other being that of Nese et al. We found that 29% (2/7) of tumors exhibited malignant behavior; however, the small number of cases with follow-up data may not have been enough to accurately determine the incidence of malignant behavior, despite our decision to minimize selection bias for malignant cases by studying consecutive cases from a single institution. At least, our findings raise an important question: is pure renal epithelioid-AML a malignant subset of AML? A review of published cases of renal epithelioid-AML yielded 66 cases of renal epithelioid-AML with a pure epithelioid component, the mortality rate from these tumors being 28.3% [7]. To prevent recurrence and receive more appropriate therapy for patients, it is important to clarify the true behavior of pure renal epithelioid-AML.

Several large studies have reported morphological indicators of tumor recurrence and metastasis [3, 4, 7]. Comparing the features of the tumors that exhibited malignant behavior with those in which recurrence and metastasis did not occur (Table 1), the following clinicopathological features occurred more frequently in those with malignant behavior: (i) local invasion; (ii) high mitotic count (> one mitosis/50 HPFs) and atypical mitotic figures; (iii) high percentage of multinucleated giant cells; and (iv) mutant p53 immunostaining. The first two of these features are consistent with the findings of Nese et al. and Brimo et al. [4, 7], and are important indicators of malignant biological behavior. The last two features suggest that mutant p53 expression and a high percentage of multinucleated giant tumor cells are probably also predictors of an adverse prognosis. Multinucleated giant tumor cells in renal epithelioid-AMLs may result from incomplete division of tumor cells and thus represent a type of dedifferentiation. In our study cohort, a high percentage of multinucleated giant tumor cells was often accompanied by increased atypia and higher mitotic counts. To our knowledge, this is a new finding, the prognostic value of which we expect to be validated in the future. Mutant p53 expression is a reliable diagnostic adjunct and adverse prognosis indicator for many tumors, such as ovarian and endometrial carcinoma, uterine leiomyosarcoma, urothelial carcinoma, gastric carcinoma, and high-grade B-cell lymphoma. There are no large series of studies about the relationship of between mutant p53 expression and prognosis of renal epithelioid-AML so far. Only a few case reports had been published [12–16]. One of five cases of renal epithelioid-AML reported by Park et al. [13] exhibited malignant behavior, this tumor had strong p53 immunoreactivity. Li et al. and Kawaguchi et al. have each reported one case of metastatic renal epithelioid-AML with strong p53 staining [14, 16]. Statistical analysis of the current series of consecutive cases showed that mutant p53 expression was correlated with poor prognosis, indicating that tumors with mutant p53 expression may have malignant behavior. p53 aberration may participate in the malignant transformation of renal epithelioid-AML. At present, there are no markers to predict the malignant behaviors of renal epithelioid-AML at initial presentation; the diagnosis of malignant epithelioid-AML often is made after metastasis/recurrence. p53 is potentially a useful and interesting prognostic indicator for renal epithelioid-AML; this possibility warrants further investigation by larger studies. Of note, our two consultation cases did not have mutant p53 expression but behaved malignantly, indicating that not all malignant renal epithelioid-AML have mutant p53 expression. Other aberrations may play a role in the malignant transformation of renal epithelioid-AML.

*TFE3* gene abnormalities have been described in some renal epithelioid-AMLs. Most of them were *TFE3* gene rearrangements and a few *TFE3* gene amplification. According to the available follow-up data, half of renal epithelioid-AMLs with *TFE3* gene rearrangement have aggressive clinical courses [17–24], suggesting that these comprise a more aggressive subset. Thus far, three cases of TFE3*-*positive renal epithelioid-AMLs with *TFE3* gene amplification have been reported [25–27]. In the present study, three cases were strongly positive for TFE3 (positivity rate 21%). However, no *TFE3* gene rearrangement was found in the three TFE3-positive cases or any of the TFE3-negative cases. This suggests that *TFE3* gene rearrangement in renal epithelioid-AMLs is very rare and that being positive for TFE3 protein does not mean that there is *TFE3* gene rearrangement in tumor cells. Two of these three cases were found to have *TFE3* gene amplification in tumor cells despite the absence of split signals. *TFE3* gene amplification should be distinguished from multiple signals in multinucleated giant tumor cells. In the tumor without *TFE3* gene amplification in this study, multiple signals appeared to be in singly scattered multinucleated giant tumor cells but not in all tumor cells; the cells with multiple signals were larger than ordinary tumor cells. *TFE3* gene amplification should also be discriminated from X chromosome polysomy in neoplastic cells. The two consultation cases in this study were found to evidence true *TFE3* amplification by a centromeric probe, which showed 2 X chromosome centromeric signals in each neoplastic cell. These two cases were found to have local recurrence/metastasis 7 months and 8 months postoperatively. Argani et al. first reported a lung metastasis of earlier uterine PEComa with *TFE3* gene amplification [28]. Since then, three renal epithelioid-AMLs with *TFE3* gene amplification have been reported, all of which developed recurrence and metastases within one year after surgery [25–27]. Both the two cases in this study and the published cases reveal that epithelioid-AMLs with *TFE3* gene amplification behave aggressively. *TFE3* gene amplification appears to be another adverse predictor for renal epithelioid-AMLs. Including the two consultation cases in this study, all epithelioid-AMLs with *TFE3* gene amplification were female and all had poor prognoses with tumor recurrence/metastasis within a year of surgery, raising the probability that epithelioid-AMLs with *TFE3* gene amplification may constitute a distinctive subset of PEComa.

This study has a few noteworthy limitations. Although this is a series study of pure epithelioid-AMLs of the kidney, having the low case number was not avoided, partially because of the rarity of these tumors. Additionally, the patients could be followed up only by phone calls, which led to a considerable proportion of them being lost to be follow-up.

Renal epithelioid-AMLs may be very similar to a variety of epithelial tumors occurring in the kidney. Differentiating them from clear cell renal cell carcinomas is challenging. Cathepsin K, HMB-45, and Melan-A can help in distinguishing renal epithelioid-AMLs from clear cell renal cell carcinomas. In addition to histomorphology, the immunophenotype of renal epithelioid-AML overlaps with MiT family renal cell carcinoma with *TFE3* or *TFEB* translocation. *PAX8* and *CD68* (PG-M1) are useful in differentiating them: *CD68* (PG-M1) is reportedly positive and *PAX8* negative in renal epithelioid-AMLs, whereas MiT family renal cell carcinoma is negative for *CD68* (PG-M1) and positive for *PAX8* [29, 30]. Furthermore, the myogenic markers smooth muscle actin and calponin are reportedly expressed in varying degrees in renal epithelioid-AML, whereas none of the MiT family renal cell carcinomas express these myogenic markers. The difference between renal epithelioid-AML and pigment Xp11 renal neoplasms is the presence of melanin pigment in the latter [24].

In summary, our relatively small series from a single institution presents the incidence of malignant behavior in pure epithelioid angiomyolipomas. We focused on the prognostic value of aberrant p53 expression and found that mutant p53 expression may be an adverse prognostic indicator for renal epithelioid-AML. *TFE3* gene rearrangement is very rare in renal epithelioid-AML, and the few cases with *TFE3* gene amplification have poor prognoses.

## Abbreviations

AML: Angiomyolipoma
Epithelioid-AML: Epithelioid angiomyolipoma
HMB-45: Human melanoma black-45
HPF: High power field
PEComa: Perivascular epithelioid cell tumor
FISH: Fluorescence in situ hybridization
